# Post-intubation hypotension and its associated factors among geriatric patients who undergo surgery at Eastern Ethiopia referral hospitals: multi-center cross-sectional study

**DOI:** 10.1097/MS9.0000000000005456

**Published:** 2026-08-27

**Authors:** Bekele Buli, Tagesse Taye, Firomsa Bekele, Bayisa Garbessa

**Affiliations:** a Department of Anesthesia, College of Medicine and Health Sciences, Hawassa University, Hawassa, Ethiopia; b Department of Anesthesia, College of Medicine and Health Sciences, Wollega University, Nekemte, Ethiopia; c Department of Anesthesia, College of Medicine and Health Sciences, Dire Dawa University, Dire Dawa, Ethiopia

**Keywords:** anesthesia, frailty, geriatrics, hypotension, post-intubation, preloading, Magnitude, Factors

## Abstract

**Background:**

Post-intubation hypotension refers to a marked drop in blood pressure after endotracheal intubation, which can result in serious complications, especially in elderly patients. The precise magnitude and contributing factors of post-intubation hypotension in geriatric anesthesia practice remain unclear. Therefore, further investigation is required.

**Objective:**

To determine the magnitude and factors associated with post-intubation hypotension among geriatric patients who underwent surgery under general anesthesia at the Eastern Ethiopia Referral Hospital in 2023.

**Method:**

A multi-center cross-sectional study was conducted on geriatric patients who underwent surgery under general anesthesia with endotracheal intubation. A systematic random sampling technique was used to select participants. Data were collected using semi-structured questionnaires that included sociodemographic information, clinical conditions, surgical factors, and anesthesia-related factors. Associations between baseline characteristics and outcome variables were assessed using binary logistic regression. Variables with *P*-values < 0.05 in multivariable logistic regression were considered statistically significant.

**Results:**

The magnitude of post-intubation hypotension in our study was 36.1%. Being extremely old age (AOR = 3.1, CI = 1.30–7.29), having a higher American Society of Anesthesiologists (ASA) physical status (AOR = 5.5, CI = 2.57–11.70), low baseline blood pressure (AOR = 4.8, CI = 2.56–8.95), and absence of fluid administration before induction (AOR = 2.9, CI = 1.41–6.29) were independently associated with post-intubation hypotension.

**Conclusion and recommendations:**

Based on our data, the magnitude of post-intubation hypotension in geriatric surgical patients is notably high. Anesthetists must be aware that endotracheal intubation of an extremely old age, ASA class III patient, pre-existing hypotension, and intubating the patient without preloading fluid were associated with post-intubation hypotension.

## Introduction

As the global population ages, the number of geriatric patients undergoing major surgical procedures is rising^[^1^]^. Despite significant advances in anesthetic management, the perioperative period remains challenging, especially in geriatric patients ^[^2^]^. These patients are highly susceptible to hemodynamic instability, with post-intubation hypotension (PIH) being one of the most critical and commonly encountered complications^[^3,4^]^.

HIGHLIGHTS

- Post-intubation hypotension is a common and serious complication among geriatric surgical patients.
- Advanced age, a higher ASA status, low blood pressure before propofol induction for intubation, and the absence of fluid administration before induction were predictors.
- Individualized anesthesia approaches are required for perioperative safety in geriatric patients.

Various definitions of PIH have been reported. It commonly refers to a sudden drop in systolic blood pressure (SBP) to<90 mmHg that occurs shortly after induction of general anesthesia with endotracheal intubation until the start of surgery^[^5–7^]^. Many researchers use this definition as the standard in critical care for the geriatric population^[^7^]^. PIH might also be defined as a mean arterial pressure (MAP) <65 mmHg or a decrease in blood pressure of at least 20% from baseline, requiring vasopressors^[^6,8^]^.

In contrast to younger individuals, geriatric patients possess a limited hemodynamic range, in which even short-lived episodes of hypotension can result in serious tissue hypoxia, organ dysfunction, neurological issues, and shock^[^9^]^. Their diminished ability to regulate vascular function makes them more vulnerable to fluctuations in blood pressure, and they may experience hypotension, demonstrating an increased sensitivity to anesthetic agents^[^6,10,11^]^.

The magnitude of PIH varies across studies, ranging from 25% to as high as 65% in older populations^[^12,13^]^. Some researchers consider it a rare complication, whereas others argue that it is fairly common. This variability is explained by the differing definitions of post-intubation hypotension used in numerous studies and also depends on the patient’s baseline health, surgical and pharmacological protocols, and, mainly, anesthetic drugs^[^5,11,14^]^.

The studies identified three factors (both modifiable and non-modifiable) that are related to PIH in the general population. These factors are patient-related, anesthetic-related, and surgical-related factors. Advanced age, male sex, coexisting medical illness, higher American Society of Anesthesiologists (ASA) score, frailty score, baseline blood pressure, emergency surgery, and induction agents were significantly associated with an increased risk of PIH^[^7,12,13,15–17^]^.

Although a few studies have explored this issue worldwide, the actual magnitude of post-intubation hypotension and its contributing factors in the geriatric population have not yet been investigated in this setting. Understanding these factors will help anesthesiologists anticipate hemodynamic instability, apply timely preventive measures, and ultimately improve the safety of elderly patients undergoing general anesthesia. Therefore, this study aimed to determine the magnitude of post-intubation hypotension and its associated factors among geriatric patients undergoing surgery under general anesthesia.

## Methods and materials

### Study design and setting

A multicenter cross-sectional study was conducted at three Eastern Ethiopia Referral Hospitals from 1 December 2022 to 30 April 2023 after obtaining ethical approval from the Ethical Review Board (IRB no.: DDU-2022-089), Dire Dawa University. This study was reported according to the STROCSS criteria from www.strocssguideline.com^[^18^]^.

### Population

#### Sources of population

All geriatric patients (aged ≥65 years) underwent elective surgery under general anesthesia at referral hospitals in Dire Dawa University.

#### Study population

This study included all eligible geriatric patients who underwent elective surgery with endotracheal intubation during the study period.

### Eligibility criteria

#### Inclusion criteria

- Age ≥ 65 years
- Undergoing elective surgery under general anesthesia with endotracheal intubation.
- Patients with ASA physical status I–III.

#### Exclusion criteria

- Patients who were undergoing emergency surgery.
- Patients were undergoing surgery with a face mask or a laryngeal mask airway.
- Patients in whom difficult intubation was anticipated.
- Patients received combined general and neuraxial anesthesia.
- Patients who were taking antihypertensive drugs, vasopressors, or inotropic infusions before induction of general anesthesia were excluded.

### Sample size determination and sampling technique

The sample size was calculated using the single-population proportion formula, assuming a 95% confidence level and a 5% margin of error. As no previous studies had been conducted in the study area or in similar African settings, a proportion (*P*) of 0.5 was used to obtain the maximum sample size. In addition, a 10% non-response rate was considered. The final sample size was 422 participants.

Participants in the study were chosen from the daily operation schedule list using the systematic random sampling technique. According to the number of elderly patients scheduled for geriatric surgery, 844 patients were treated over the course of 3 months at three different hospitals, according to the logbook. To allocate the sample proportionally, the number of geriatric patients in each hospital was divided by the total number of geriatric patients across all sites and then multiplied by the total sample size (*n* = 422), as shown in Figure 1

**Figure 1.**
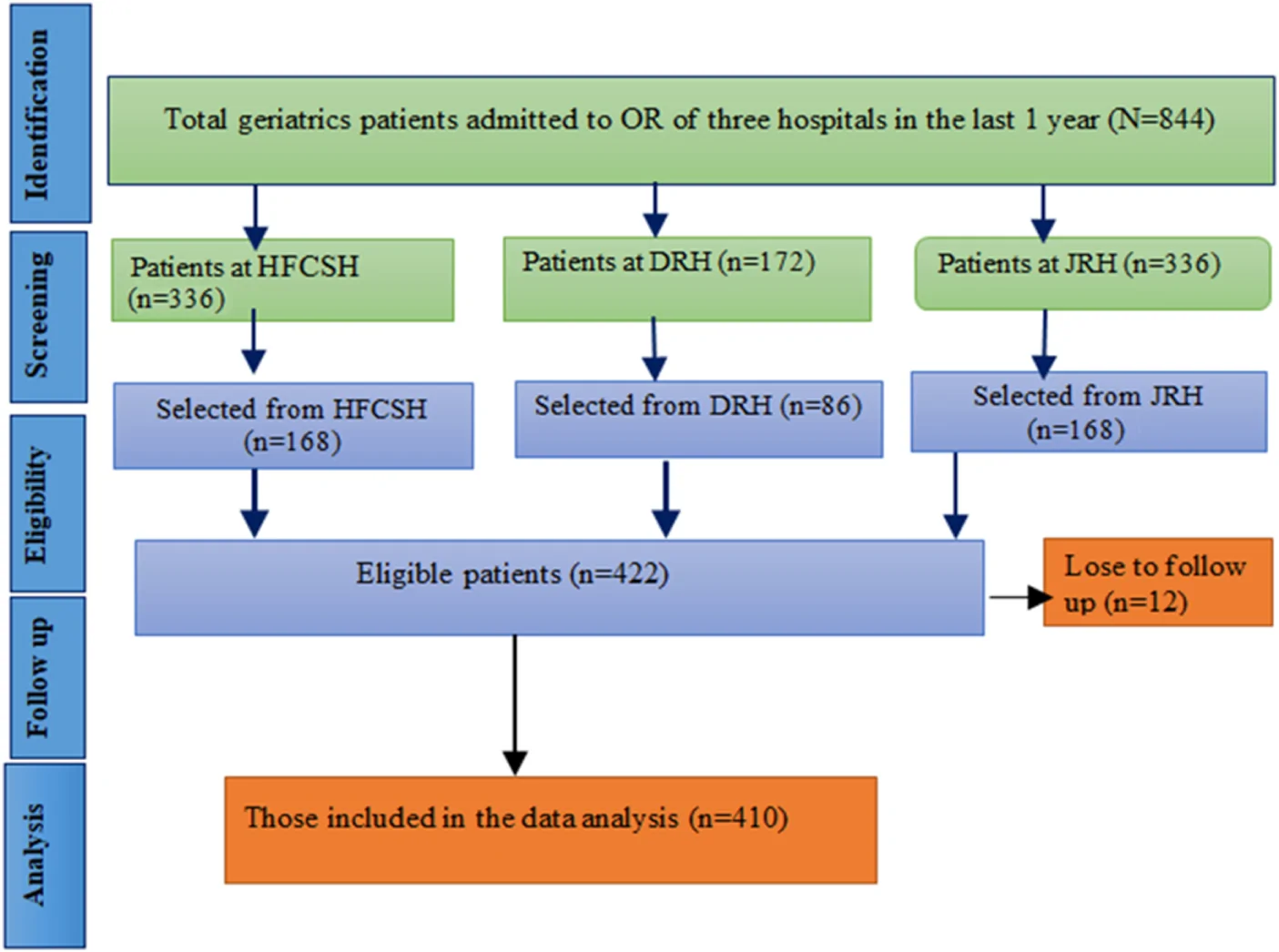
STROBE flow chart of geriatric patients admitted to the OR in Eastthen Ethiopia referral hospitals.

The formula *K* = *N*/*n*, where n is the total sample size and *N* is the population size, was used to calculate the sampling interval *K*. A recruitment rate of 422 participants was achieved with a 50% chance. Consequently, the sampling interval was two, and the data collector recruited one patient out of every two successive patients after selecting the first research participant using a lottery method.

### Variables

#### Dependent variables

- Post-intubation hypotension (PIH).

#### Independent variables

- Socio-demographic characteristics: age, sex, and BMI
- Patient-related factors: ASA status, frailty score, and vital signs.
- Anesthesia-related factors include the induction agent used, analgesic drugs used, premedication, the number of ETI attempts, and fluid administered before intubation.
- Surgery-related factors: types of surgery (General, Urology, Neurology, Orthopedics, Gynecology, and ENT surgeries).

### Data analysis

The data were checked manually for completeness, coded, entered into Epi Info version 7, and then exported to SPSS version 26 for analysis. Descriptive statistics were used to summarize the data, and the results are presented in tables and figures. Continuous variables were expressed as the mean ± standard deviation for normally distributed data and as the median with interquartile range for skewed data. Selected continuous variables were categorized for analytical purposes.

Frequencies and cross-tabulations were performed to describe the distribution of variables in relation to the outcome. Binary logistic regression analysis was conducted to identify the factors associated with the outcome variable. Variables with a *P*-value < 0.25 in the bi-variable analysis were entered into the multivariable logistic regression model^[^19^]^. In the multivariable analysis, variables with a *P*-value < 0.05 were considered statistically significant.

### Data collection techniques

Data were collected using a structured questionnaire developed from previous studies (see Supplemental Digital Content file, available at: https://links.lww.com/MS9/B346). The tool included variables such as socio-demographic characteristics, ASA physical status, type of surgery, anesthetic agents used, number of intubation attempts, and pre- and post-intubation blood pressure measurements. In this study, post-intubation hypotension was defined as a SBP<90 mmHg within the first 20 minutes after induction of anesthesia and before surgical incision; therefore, blood pressure was recorded at 5-minute intervals during this period. In the study hospitals, the choice of induction technique followed local practice and was determined by the anesthetist in charge.

### Data quality control

Data were collected using a pretested self-administered structured questionnaire and checklists. A pretest was conducted with 5% of the sample population, and necessary amendments were made. Regular supervision and follow-up were conducted during data collection.

### Operational definitions

- Geriatrics: patients aged 65 and older.
- Frail: patients who had scored > 4 on the clinical frailty score^[^20^]^
- Normal baseline BP: SBP of 120–139 mmHg and diastolic BP of 80–90 mmHg^[^21^]^
- Intubation attempt: the number of attempts required to successfully insert an ETT into the trachea
- Post-intubation hypotension: a drop in SBP to <90 mmHg occurring shortly after endotracheal intubation and before surgical incision^[^5^]^

### Ethical considerations

Ethical clearance was obtained from the Institutional Review Board (IRB) of Dire Dawa University under reference number DDU-2022-089. This study was conducted in accordance with the Declaration of Helsinki. Permission letters were obtained from the participating hospitals. Confidentiality was maintained, and informed consent was obtained.

## Results

### Socio-demographic and clinical characteristics

A total of 410 geriatric patients were included in this study, yielding a response rate of 97%. Of these, the majority were female (214 [52.2%]), and most were aged 65 to 70 years (48.8%), as demonstrated in Table 1.

**Table 1:** Socio-demographic associated factors among geriatric patients undergoing surgery with general anesthesia at Eastthen Ethiopia referral hospitals, from 1 December 2022 to 30 April 2023 (*N* = 410).

Variables	Category	Frequency (%)
Gender	Female	214 (52.2)
	Male	196 (47.8)
Age (years)	65-70	200 (48.8)
	70-75	106 (25.9)
	75-80	46 (11.2)
	>80	58 (14.1)
Body mass index (kg/m^2^)	< 18.5	25 (6.1)
	18.5-24.9	295 (72.0)
	>24.9	90 (22.0)
ASA physical status	ASA I	233 (56.8)
	ASA II	97 (23.7)
	ASA III	80 (19.5)
Baseline blood pressure status	Normal*	139 (33.9)
	Low	227 (55.4)
	High	44 (10.7)
Frailty score	Frail	187 (45.6)
	Non frail	223 (54.4)

ASA: American Society of Anesthesiologist, *systolic and diastolic blood pressure for older adults 120–139 mmHg and 80–89 mmHg, respectively

### Anesthetist-related and surgery-related factors

The study showed that most participants were not premedicated for anxiety, 363 (88.5%), and succinylcholine was the most widely used muscle relaxant for intubation. The most frequent surgical category during the study period was general surgery, 106 (25.9%), as shown in Table 2

**Table 2:** Anesthetics-related factors among geriatric patients undergoing surgery with general anesthesia at Eastthen Ethiopia referral hospitals, from 1 December 2022 to 30 April 2023 (*N* = 410).

Variables	Category	Frequency (%)
Anti-anxiety	No	363 (88.5)
	Yes	47 (11.5)
Analgesic drugs	Morphine	158 (38.5)
	Fentanyl	65 (15.9)
	Pethidine	187 (45.6)
Induction drugs	Ketamine	172 (42.0)
	Thiopental	109 (26.6)
	Propofol	129 (31.4)
Muscle relaxants	Succinylcholine	293 (71.5)
	Vecuronium	117 (28.5)
Inhalational agents	Isoflurane	271 (66.1)
	Halothane	139 (33.9)
Fluid before induction	>500 ml	186 (45.3)
	<500 ml	134 (32.7)
	No fluid	90 (22.0)
Intubation attempts	Single	277 (67.6)
	Multiple	133 (32.4)
Types of surgery	General	106 (25.9)
	Orthopedics	95 (23.2)
	Urology	75 (18.3)
	Neurology	64 (15.6)
	Gynecology	40 (9.8)
	ENT	30 (7.3)

### Magnitude of PIH

In this study, the overall incidence of post-intubation hypotension among geriatric patients who underwent surgery under general anesthesia was found to be 36.10% (95% CI: 31.2–40.9) in Eastern Ethiopia referral hospitals, as shown in Figure 2.

**Figure 2.**
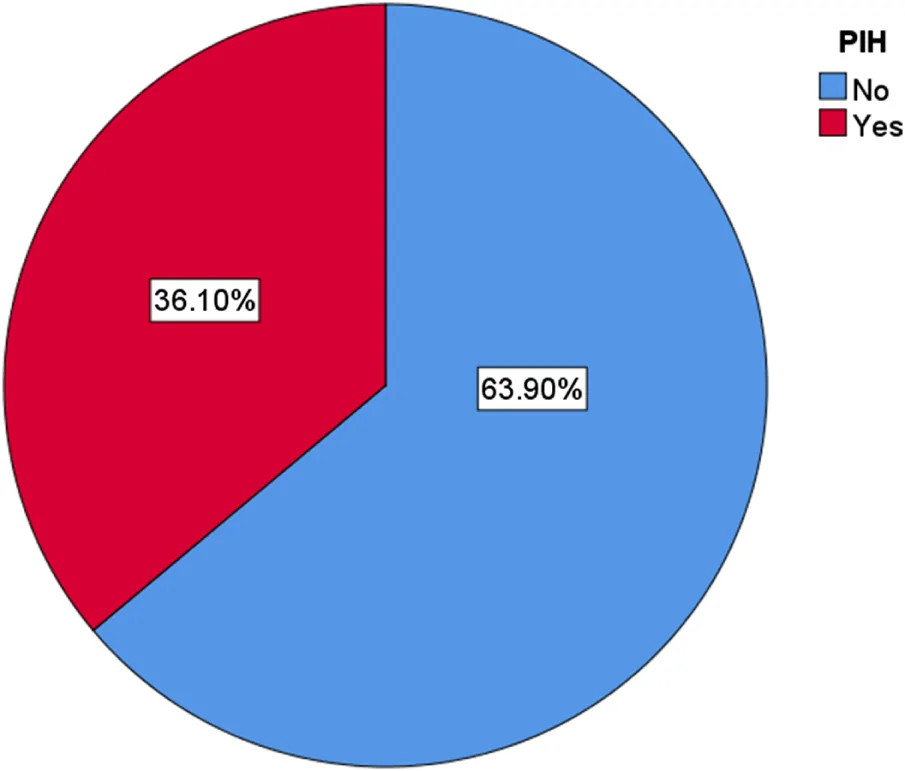
The magnitude of post-intubation hypotension at Eastthen Ethiopia referral hospitals (EERHs).

### Bivariate and multivariate logistic regression analyses

Binary logistic regression analysis was used to identify the association between the independent and outcome variables. Variables with a *P*-value less than 0.25 in the bivariate logistic analysis were considered candidates and were entered into the multivariate logistic analysis using the forward Wald selection method. In the multivariate logistic analysis, variables with a *P*-value less than 0.05 were considered statistically significant. The model of fit test was assessed using the Pearson chi-square test (χ^2^ = 147.982, df = 9, *P* < 0.001) and the Hosmer–Lemeshow test (χ^2^ = 14.832, df = 8, *P* = 0.162). The insignificant result of the Hosmer–Lemeshow test revealed that the model has good goodness of fit. The pseudo *R*^2^ was 0.513, indicating that the model explained approximately 51.3% of the variability in PIH. The remaining 48.7% was explained by other factors.

The variables associated with post-intubation hypotension in the bivariate logistic regression with *P*-values less than 0.25 include age, ASA Physical Status, pre-induction blood pressure, preoperative fluids administered, frailty score, premedication for anxiety, and induction agents.

In the multivariable logistic regression analysis, age >80 + years, ASA physical status III, low pre-induction blood pressure, and not receiving preoperative fluid were significantly associated with PIH with a *P*-value of 0.05. Patients aged >80 years were three times more likely to develop PIH compared with those aged 65–70 years (AOR = 3.1, 95% CI: 1.30–7.29, *P* = 0.010). Patients with ASA physical status III were 5.5 times more likely to develop PIH compared with those with ASA physical status I (AOR = 5.5, 95% CI: 2.57-11.70, P = 0.0001). The other variables were reported in Table 3.

**Table 3:** Factors associated with PIH among geriatric surgery patients at Eastern Ethiopia referral hospitals from 1 December 2022 to 30 April 2023 (n = 410).

Variables	Category	PIH		Bivariate analysis		Multivariate analysis	
		No (%)	Yes (%)	COR (95%CI)	*P*-value	AOR (95% CI)	*P*-Value
Age (in Years)	65–70	148 (56.5)	52 (35.1)	1		1	
	70–75	78 (29.8)	28 (18.9)	1.0 (0.59–1.75)	0.937	0.8 (0.0.42–1.44)	0.422
	75–80	22 (8.4)	24 (16.2)	3.1 (1.61–6.00)	0.001	1.6 (0.74–3.62)	0.222
	>80	14 (5.3)	38 (25.7)	8.9 (4.50–17.64)	0.0001	**3.1 (1.30-7.29)**	0.010*
ASA status	I	176 (67.2)	57 (38.5)	1		1	
	II	68 (26.0)	29 (19.6)	1.3 (0.78–2.23)	0.306	1.4 (0.74–2.49)	0.314
	III	18 (6.9)	62 (41.9)	10.6 (5.82–19.45)	0.0001	**5.5 (2.57–11.70)**	0.0001*
Frailty score	No Frail	135 (51.5)	52 (35.1)	1	1	1	1
	Frail	127 (48.5)	96 (64.9)	1.9 (1.30–2.97)	0.001	1.2 (0.61–2.71)	0.245
Baseline BP	Normal	121 (46.2)	18 (12.2)	1		1	
	Low	109 (41.6)	118 (79.7)	7.3 (4.16–12.73)	0.0001	**4.8 (2.56–8.95)**	0.0001*
	High	32 (12.2)	12 (8.1)	2.5 (1.10–5.78)	0.029	0.7 (0.26–2.0)	0.535
Fluid given	>500 ml	140 (53.4)	46 (31.1)	1		1	
	<500 ml	94 (35.9)	40 (27.0)	1.3 (0.79–2.13)	0.309	0.8 (0.46–1.51)	0.548
	No fluid	28 (10.7)	62 (41.9)	6.7 (3.9–11.76)	0.0001	**2.9 (1.41–6.29)**	0.004*
Anti-anxiety	No	236 (90.1)	127 (85.8)	1	1	1	1
	Yes	26 (9.9)	21 (14.2)	1.5 (0.81-2.77)	0.193		
Induction agents	Ketamine	113 (43.1)	59 (3.9)	1	1	1	1
	Thiopental	83 (31.7)	26 (17.6)	0.6 (0.35–1.03)	0.064	1.5 (0.65–1.67)	0.781
	Propofol	66 (25.2)	63 (42.6)	1.8 (1.15–2.92)	0.011	0.7 (0.35–1.67)	0.423

PIH: Post Intubation Hypotension, 1: Reference group,*: statistically significant, Variables presented in bold were statistically significant, CI: Confidence Interval

## Discussion

In our study, the overall magnitude of PIH among geriatric patients who underwent surgery was 36.10%. This highlights that more than 1 in 3 geriatric patients experienced a critical drop in blood pressure immediately after intubation. This finding underscores a significant clinical challenge in the anesthetic management of geriatric patients in our multi-center study, where real-time advanced hemodynamic monitoring was limited.

This finding was consistent with previous studies that generally reported that PIH rates were between 25% and 65% in various clinical settings^[^22–26^]^. However, our results were higher than those of previous studies^[^5,27^]^. This difference may be due to variations in definitions (in our study, PIH was defined using the SBP threshold), clinical settings, and socio-demographic factors.

Our study identified extreme old age (>80 years) as a potent predictor, with these patients being three times more likely to experience PIH than those aged 65–70 years. This is likely due to age-related reductions in physiological reserves, reduced autonomic regulation, or a diminished baroreceptor reflex response, which impair patients’ ability to compensate for anesthetic effects. Similarly, previous studies from Thailand, Canada, and India have reported that extreme old age was independently associated with the occurrence of post-intubation hypotension^[^12,23,28^]^.

Additionally, our findings demonstrated that higher ASA physical status (III) was a factor associated with PIH. This finding is consistent with studies by Jor *et al* and Reich *et al*, which showed that higher ASA physical status was a risk factor for PIH ^[^3,24^]^. This may be because patients with higher ASA physical status lack the compensatory mechanisms required to handle the sympatholytic effects of induction agents.

Another significant factor was pre-existing low blood pressure before intubation, which was strongly associated with the development of PIH. Our study is similar to a study conducted in China by Cheng *et al*., which showed that baseline SBP was independently associated with PIH^[^29^]^. This supports the idea that lower baseline blood pressure (due to intravascular hypovolemia, preoperative fasting or dehydration, and preexisting disease) is an important predictor of PIH^[^3,13^]^.

Lastly, in our study, we found that not administering fluids before induction was significantly associated with a higher incidence of post-intubation hypotension compared with patients who received pre-induction fluids. Our findings align with those of previous studies, which showed a significant increase in the incidence of PIH among patients who did not receive pre-intubation fluids compared with those who did^[^30,31^]^.

In contrast to other studies, sex, BMI, frailty status, anesthetic medications, and surgical type did not show significant associations with PIH in our study^[^5,13,14,22,32^]^. This might be due to differences in the study populations and settings. Regarding anesthetic drugs, we observed that the selection of an induction agent was not associated with PIH, which is inconsistent with studies conducted in Croatia and Thailand that found induction with propofol to be the most significant factor associated with post-intubation hypotension^[^16,23^]^. A possible explanation for this difference might be that the most widely used induction agent in those studies was ketamine, at around 42%.

Generally, our results indicate that individual patients' baseline characteristics, along with anesthesia practice, play a crucial role in the occurrence of post-intubation hypotension. These factors are essential for assessing risk and developing targeted prevention strategies to enhance hemodynamic stability in this population.

### Strength of the study

Its strength lies in the multi-center design and systematic sampling technique, which enhance the generalizability of the results within the local region. Data were collected from a multidisciplinary institution, encompassing various units involved in geriatric surgery.

### Limitations of the study

A notable limitation of this study is the scarcity of comparable literature on this topic within a similar context, making it challenging to align our findings with those of previous studies. Excluding patients on medications and those undergoing emergency surgery may underestimate the real-world burden of PIH, especially among higher-risk geriatric populations. Additionally, we did not collect data on potential long-term complications that might arise after patients left the operating room. Given that the study design was observational and cross-sectional, it captured only a snapshot in time, preventing any cause-and-effect conclusions.

## Conclusion

This study revealed that the magnitude of post-intubation hypotension is a highly prevalent complication among geriatric patients in Eastern Ethiopian referral hospitals, affecting more than a quarter of the study population. The findings underscore that hemodynamic instability is not a random occurrence but is significantly influenced by both patient-specific and provider-dependent factors. Therefore, individualized anesthesia induction methods for elderly patients may require tailored strategies to ensure perioperative safety.

### Recommendation

The recommendtion was based on the findings of the present study.

### For anesthesia providers

- The magnitude of PIH was high in this study; therefore, consider fluid loading for geriatric patients with advanced age, high ASA scores, and low baseline blood pressure before induction and tracheal intubation.

### For the hospitals/institutions

- Develop standard protocols for geriatric guidelines specific to induction methods..
- Ensure the consistent availability of vasopressors and fluids in all operating rooms..

### For researchers

- Future research should utilize prospective cohort designs or randomized controlled trials to evaluate the long-term impact of PIH on post-operative outcomes in the Ethiopian context.

## Data Availability

The data that support the findings of this study are available upon reasonable request.

## Research registration unique identifying number (UIN)

researchregistery0000.
